# Pediatric scorpionism in northern Amazonia: a 16-year study on epidemiological, environmental and clinical aspects

**DOI:** 10.1590/1678-9199-JVATITD-2020-0038

**Published:** 2020-09-11

**Authors:** Jules Vaucel, Remi Mutricy, Maëlle Hoarau, Jean-Marc Pujo, Narcisse Elenga, Magali Labadie, Hatem Kallel

**Affiliations:** 1 Poison Control Center (Centre Antipoison) Nouvelle Aquitaine, Centre Hospitalier et Universitaire Pellegrin, Bordeaux 33076, Aquitaine, France.; 2 Emergency Department, Centre Hospitalier de Cayenne, Cayenne 97300, French Guiana, France.; 3 Emergency Pediatric Department, Centre Hospitalier de Cayenne, Cayenne 97300, French Guiana, France.; 4 Intensive Care Unit, Centre Hospitalier de Cayenne, Cayenne 97300, French Guiana, France.

**Keywords:** Scorpion, Scorpion sting, Tityus obscurus, Pediatric emergency medicine, Intensive care units, Pediatric

## Abstract

**Background:**

The Amazon basin is one of the seven major geographical areas where scorpionism is recorded. In French Guiana, 90 stings per 100,000 inhabitants are registered per year. As the severity of cases is higher in children, descriptive studies are needed to have a better understanding of this pathology. The aim of the present study is to describe pediatric scorpionism in French Guiana.

**Methods:**

We conducted a monocentric descriptive retrospective study on scorpion stings in all pediatric patients admitted to Cayenne General Hospital from January 1, 2002 to December 31, 2018.

**Results:**

In this survey, 132 patients were included. Of them, 63% were male. Patients with general signs of envenomation were younger and lighter (p = 0.04). The picture was “one sting” (95.3%) by a “big” (47.6%), “black” (60%) and “small pincer” (58%) scorpion on the extremity of the body (84%). Stings occurred mainly during the day, while patients changed clothes. There was no envenomation during night. The monthly evaluation highlights that the number of stings and percentage of general signs of envenomation were closely connected to a composite variable including the variation of the level of rivers (p = 0.005). Cardiac symptoms were recorded in 82% of cases with general signs of envenomation. The presence of pulmonary; ear, nose, and throat (ENT); or gastrointestinal symptoms are related to major envenomation (p = 0.001, p = 0.01, and p = 0.02 respectively). Leukocytosis and glycemia increased according to the envenomation grade whereas serum potassium and alkaline reserve decreased. Forty-six patients needed hospitalization and seven of them required intensive care. No patient died nor presented sequelae at discharge from the hospital.

**Conclusion:**

Pediatric scorpionism in French Guiana is closely associated with child activities and climatic conditions. Severe envenomation presented most of the time with cardiac, pulmonary, and gastrointestinal symptoms.

## Background

The Amazon basin is one of the seven major geographical areas with a high incidence of scorpion stings. Thus, 28.5 stings per 100,000 inhabitants are recorded worldwide every year and 0.08% of them results in death [1]. Previous studies have shown that the incidence of scorpion stings is closely related to climatic conditions [2]. Indeed, in Amazonia, scorpion stings are more common during the rainy season [3-5] differing from the other major regions where scorpion stings occurred during the dry season [6-8]. It is noteworthy that the *incidence* of scorpion stings is underestimated because most patients do not consult their physicians after the sting [9]. Besides that, pediatric scorpionism remains a public health problem due to the high incidence and potential severity [10]. 

In French Guiana, 30 scorpion species have been identified and their distribution is well known [11]. Only three species are responsible for severe stings: *Tityus obscurus*, *Tityus silvestris*, and *Isometrus maculatus* [12,13]. These three species are from the Buthidae family and are considered “opportunistic” [14]. They are present throughout the territory and notably in the city [11], and are responsible for 90 stings per 100,000 inhabitants/year [15]. In addition, the two deaths recorded in French Guiana since 1997 caused by scorpion envenomation were children stung by *Tityus obscurus* [16,17]. In French Guiana, children represent 27.8% of calls to the regional emergency call service (SAMU 973) and 22.1% of consultations in the emergency department for scorpion sting [3,18]. Moreover, scorpion envenomation is globally more severe for children than adults [19-21]. Therefore, we designed this study to describe the epidemiological, environmental, clinical, biological and therapeutic features of pediatric scorpionism in French Guiana.

## Methods

### Study population and environment

French Guiana is an overseas region of France located on the North Atlantic coast of South America between the third and the fifth North degree. In 2018, the population was estimated to be 296,700 inhabitants, which represents 3.5 inhabitants/km². People under 20 years old represents 41.7% of the population. Most inhabitants (86%) live on the coast or along rivers. Fifty-two percent of them live in the metropolitan area of the main city Cayenne, with a density of 574 inhabitants/km². In French Guiana, there are three emergency departments (ED) (Cayenne, Kourou, and Saint Laurent du Maroni). Cayenne General Hospital is a 742-bed health facility that provides a first-line medical care for an urban population of 150,000 inhabitants. It manages 18 remote centers for prevention and primary care of almost 50,000 inhabitants. Thereby, it is also a referral center for the largest share of the Guianese population coming from all over French Guiana as well as for border areas of neighboring countries [22]. 

French Guiana shelters a unique and important ecosystem including tropical rainforests (96% of the region), coastal mangroves, savannahs, inselbergs and many types of wetlands [11]. It is a tropical area with a short dry period and a longer rainy season. The rainy season lasts from December to June and the highest period of rainfall is from April to June, with the biggest pluviometry peak in May. Daytime temperatures are higher in the forest than on the coast, while nights are cooler. Humidity is constantly high (85%) as well as temperatures of 27°C all along the year [23].

### Study setting and design

This monocentric retrospective study was conducted in the ED of the Cayenne General Hospital, and covers a 16-year period from January 1^st^, 2003 to December 31, 2018. We analyzed retrospectively all medical files of patients under 18 years old admitted to the ED due to scorpion stings. Age groups are defined as follows: neonate (0 to 1 months), infant (1 to 23 months), child (2 to 12 years), and adolescent (13 to 18 years). The selection of medical files was based on the 10^th^ revision of the International Statistical Classification of Diseases and Related Health Problems (ICD-10). The selected codes were T63.2 (venom of scorpion), T63.8 (toxic effect of contact with other venomous animals), T63.9 (toxic effect of contact with unspecified venomous animal) or X22 (contact with scorpions). Only medical files with scorpion envenomation mentioned by the physician in charge were studied. We gathered epidemiological and clinical data, including age and gender of patients, the date and time of the sting, the site of the sting on the patient’s body, the scorpion description, and the clinical symptoms at the arrival to the ED. Topics for the syndromic assessment were:

- Cholinergic symptoms including agitation, apnea, bradycardia or tachycardia, bronchoconstriction, coma, confusion, convulsion, cyanosis, high blood pressure, hypersecretion (bronchorrhea, sialorrhea and diarrhea), lethargy, myosis or mydriasis, nausea, paresthesia, paralysis, sweating, and vomiting.
- Adrenergic symptoms including abdominal pain, acidosis, agitation, seizures, fever, headache, high or low blood pressure, hypo or hyperglycemia, hypokalemia, insomnia, mydriasis, nausea, palpitations, tachycardia, tachypnea, tremor, ventricular extrasystoles, and vomiting [24].

The severity of envenomation was assessed according to the classification of the scorpion consensus experts group [25]:

- Grade I: local manifestations.
- Grade II: minor manifestations (i.e. non-life-threatening).
- Grade III: severe manifestations (i.e. life threatening).
- Grade 0: no manifestation / dry sting (an extended classification/adaptation by this research group).

The severity of envenomation was also measured using the Poisoning Severity Score [26]. For the evaluation of pediatric risk of mortality, we used the PRISM Score [27]. The need for individual patient consent was waived by the local research ethics committee. Our database has been registered at the Commission National de l’Informatique et des Libertés (registration n. 2217032), in compliance with French law on electronic data sources.

### Statistical analysis

We designed a database with information from patients and scorpions and performed a descriptive analysis using Excel 365 and Stata® Version 15 for Windows. Results were reported as median and interquartile range (25^th^-75^th^ percentiles), mean ± standard deviation, or numbers with percentages. To compare qualitative variables, Fisher’s exact test was used. To compare quantitative variables, student’s t-test and the Mann-Whitney test were employed. The significance level was set at p ≤ 0.05. Autoregressive-moving-average model (ARIMA) with ponderation (0,0,0) [28] and the coefficient of determination (r²) were utilized to determine the link between environmental condition and number of stings or percentage of general envenomation per month. For multiple analysis, the variable was kept p < 0.5. 

Receiver operating characteristic (ROC) curves were used to evaluate the diagnostic value of quantitative variable for PRIMS II according grade of envenomation. The area under the curve was estimated by the method of Hanley and McNeill [29].

## Results

### Population description

**Figure 1.**
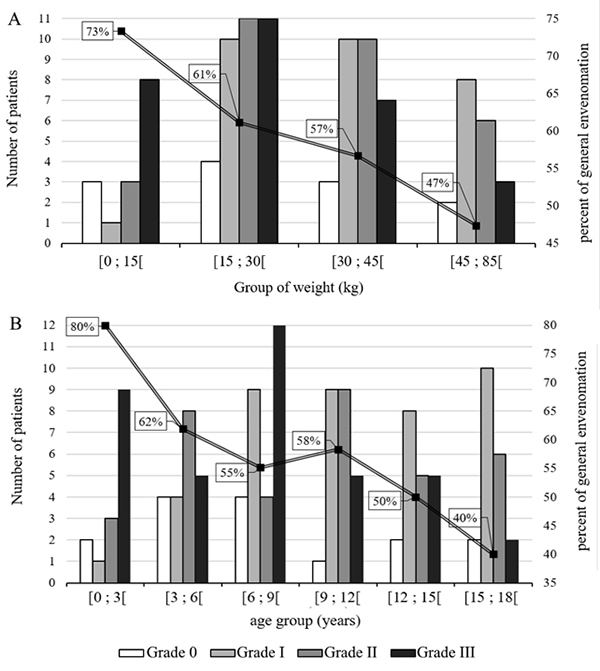
Distribution of the grade of envenomation according to patient **(A)** weight and **(B)** age. The curves represent the percentage of general envenomation by **(A)** weight and **(B)** age group.

One hundred thirty two patients stung by scorpions were finally included in the study. They were seven infants (5%), 88 children (67%) and 37 adolescents (28%). Of them, 63% were male. Fifty-seven patients (86% of the recorded values) were stung in a coastal city and the nine remaining patients (14% of the recorded values) in forest area. General signs of envenomation were present in 100% of infants, 58% of children, and 24.7% of adolescents (Figure 1B). Mean age was 10 ± 5 years in patients with Grade 0 and I envenomation and 8 ± 5 years in those with Grades II and III (p = 0.06). It was 7 ± 5 years in patients with Grade III envenomation (p = 0.0008). The weight was 28 ± 16 kg in patients with general envenomation versus 35 ± 16 kg in those without (p = 0.04) (Figure 1A). Ninety-one patients (90% of the recorded values) consulted in the first three hours after the sting without statistical difference between envenomation grades.

Patients were typically stung one time by a “big black small pincer scorpion” at home on the extremity of the body during daytime. The color of the scorpion was known in 33 cases (20%), as follows: 20 black (60%), ten brown (30%), one grey (3.3%), one yellow (3.3%) and one white (3.3%). The size of the scorpion was known in 22 cases (16%) and defined as small or less than 2.5 cm in nine cases (43%), medium or 2.5 to 5 cm length in two cases (10%), and big or more than 5 cm in 11 cases (47 %). The description of scorpions’ pincer was registered in 67 cases (58%) and described as small in 58 of them (86% of the recorded values). The number of scorpion stings was available in 126 cases (96%) as follows: one sting in 120 cases (95%), two stings in five cases (4%) and numerous stings in one case (1%). The sting site in the body was available in 125 cases (95%): 105 cases (84%) on the extremity (60 on the foot and 45 the on hand), 12 cases (10%) on the limbs (five on the arm and seven on the leg), in seven cases (5%) on the trunk and in one case (1%) on the face. Physicians in charge provided a description of the scorpion as *Tityus* in 30 cases (22%) and specified *Tityus obscurus* in 18 cases (13%).

### Environmental features at the time of the sting

**Figure 2.**
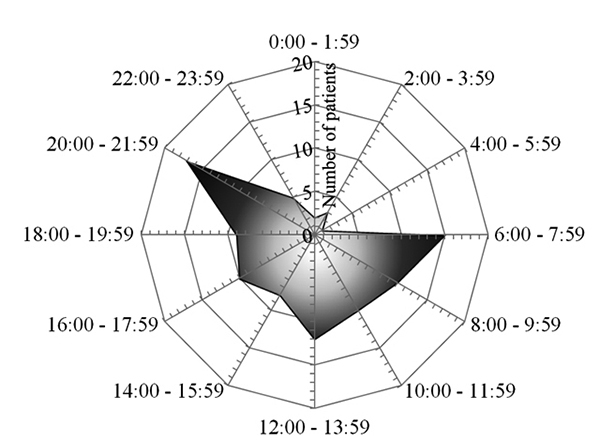
Number of patients according to the time of the day.

The circumstances of the envenomation were recorded in 58 cases (75%). Patients were at home in 48 cases (83%), at school in five cases (9%), at the beach in two cases (3%), and in forest or playground in three cases (5%). The time of the sting was recorded in 103 cases (78%) (Figure 2). The sting happened mostly during daytime (6 am to 6 pm) in 66 cases (63%), without difference between groups with and without general signs of envenomation (p = 0.36). The highest frequency of scorpion stings were observed between 06:00 and 08:00 in 15 cases (11%), 12:00 and 14:00 in 12 cases (9%), and between 20:00 and 22:00 in 17 cases (13%). 

**Figure 3.**
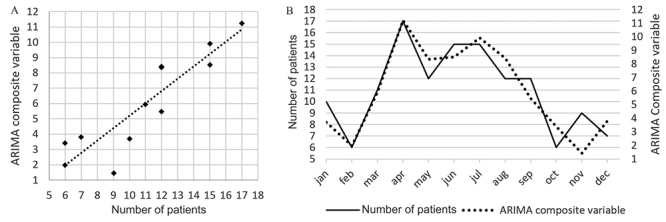
(A) Correlation between the number of patients recorded and the number predicted by the ARIMA composite variable (r² = 0.81). (B) The monthly representation of the number of stings recorded and the number predicted by ARIMA composite variable.

The distribution of cases according to the month of occurrence is reported in Figure 3B. The number of cases varied according to the rain flow, with 0.67 case/month during the dry season versus 0.70 case/month during the rainy season. It was 0.91 case/month during the highest rainy season (p = 0.02). The number of cases per month was better correlated with the water level of rivers than the other climatic variable in univariate analysis (p = 0.01 and r² = 0.40) but most with river level variation in multivariate analysis (p = 0.005) (Table 1). 

**Table 1.** Factors associated with monthly scorpion sting distribution for the study population via regression analysis per month (scorpion sting rate is response variable).

Model	Factor	b	S.E. (b)	T value	p	r^2^
UA	Water level of river	0.0465	0.020	2.35	0.01	0.40
	River flow	0.0150	0.007	2.12	0.03	0.40
	Absolute river level variation	-0.0462	0.091	0.615	0.61	0.11
	Pluviometry	0.0070	0.008	0.91	0.36	0.11
	River level variation	-0.0227	0.028	0.41	0.41	0.08
	Sunshine	-0.0002	0.017	-0.01	0.99	0.02
	Temperature	-0.4082	2.154	-0.19	0.85	<0.01
MA	Water level of river	-0.1265	0.161	-0.79	0.43	
	River flow	0.0624	0.051	1.20	0.23	
	Pluviometry	0.0049	0.006	0.77	0.44	
	River level variation	-0.0696	1.277	3.90	0.005	
	**r^2^ = 0.81, p < 0.001**					

UA: univariate analysis, MA: multivariate analysis, b: coefficient, S.E. (b): standard error of coefficient, r²: coefficient of determination

The ARIMA composite variable (Figure 3) had p <0.0001 and r² = 0.81 with mathematical formulation f(x) = 0.0625[River Flow] - 0.1264[Water level of river] + 0.0049[Pluviometry] - 0.0696[River level variation] 

**Figure 4.**
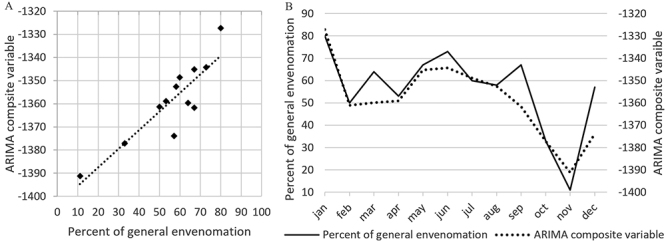
(A) Correlation between the number of general envenomation recorded and the number predicted by the ARIMA composite variable (r² = 0.77). (B) The monthly representation of the percentage of general envenomation recorded and the number predicted by the ARIMA composite variable.

The number of cases with general symptoms (i.e. Grade II or III envenomation) was unequally distributed throughout the year (Figure 4B) with the lowest number of cases in October and November (<50%) and the highest in January (80%) (p = 0.002). This percentage was better correlated with the absolute monthly river level variation (r² = 0.45, p = 0.003) than with other climatic variables but most with sunshine in multivariate analysis (p = 0.07) (Table 2). 

**Table 2.** Factors associated with monthly general envenomation distribution for the study population via regression analysis per month (general envenomation rate is response variable).

Model	Factor	b	S.E. (b)	T value	p	r^2^
UA	Absolute river level variation	-0.1261	0.1523	-0.83	0.41	0.45
	River level variation	0.0680	0.173	0.39	0.70	0.44
	Temperature	-22.0612	9.390	-2.35	0.02	0.33
	Water level of river	0.2107	0.155	1.36	0.17	0.32
	River flow	0.0688	0.049	1.40	0.16	0.31
	Pluviometry	0.0589	0.031	1.88	0.06	0.29
	Sunshine	-0.1200	0.085	-1.41	0.16	0.16
MA	Absolute river level variation	-0.3269	0.183	-1.78	0.08	
	Temperature	-54.0915	37.643	-0.27	0.15	
	Water level of river	-0.2843	1.043	-0.27	0.79	
	River flow	0.0349	0.350	0.10	0.92	
	Pluviometry	0.1451	0.857	1.69	0.90	
	Sunshine	0.5258	0.285	1.84	0.07	
	**r^2^ = 0,77 p < 0.001**					

UA: univariate analysis, MA: multivariate analysis, b: coefficient, S.E. (b): standard error of coefficient, r²: coefficient of determination

The ARIMA composite variable (Figure 4) had p <0.0001 and r² = 0.77 with mathematical formulation f(x) = -0 .3269 [Absolute river level variation] - 54.0915[Temperature] - 0.2843[Water level of river] + 0.0349 [River flow] + 0.1451[Pluviometry] + 0.5258 [Sunshine].

### Clinical pictures and paraclinical results

Patients were divided according to the Katthabi classification into 15 patients (11%) in Grade 0, 40 patients (30%) in Grade I, 38 patients (29%) in Grade II, and 39 patients (30%) in Grade III. Clinical symptoms according to the severity of envenomation are summarized in Table 3. The average number of symptoms was 3.79 in Grade III versus 1.52 in Grade II patients (p = 0.001). The average number of organs affected was 2.28 in Grade III versus 1.21 in Grade II patients (p = 0.0002). In comparison of Grade II and Grade III envenomation, tachycardia was associated with Grade II (p < 0.0001) and the presence of pulmonary or ENT symptoms (notably sialorrhea) or nausea was associated with Grade III envenomation (respectively p < 0.0001, p = 0.01, p = 0.01 and p = 0.02). 

**Table 3.** Clinical parameters recorded in the general population and according to the grade of envenomation.

	Overall population		Grade 0-I		Grade II		Grade III	
Parameter	Nb	Result	Nb	Result	Nb	Result	Nb	Result
**Local symptoms**	**131**	**104 (79.4%)**	**55**	**40 (72.7%)**	**38**	**33 (89.2%)**	**39**	**31 (79.5%)**
Pain	130	93 (71.5%)	54	35 (64.8%)	37	29 (78.4%)	39	29 (74.4%)
Paresthesia (local)	126	19 (15.1%)	54	7 (13%)	36	8 (22.2%)	36	8 (22.2%)
Erythema	128	47 (36.7%)	54	17 (31.5%)	36	14 (38.9%)	38	14 (38.9%)
Local edema	129	34 (26.4%)	54	11 (20.4%)	37	11 (29.7%)	38	12 (31.6%)
Muscle cramps	130	1 (0.8%)	55	0 (0%)	37	0 (0%)	38	1 (2.9%)
**General symptoms**	**132**	**9 (6.8%)**	**55**	**0 (0%)**	**38**	**2 (5.3%)**	**39**	**7 (17.9%)**
Fever (T > 38.5°C)	112	1 (0.9%)	49	0 (0%)	33	0 (0%)	30	1 (3.3%)
Asthenia	131	4 (3.1%)	55	0 (0%)	37	0 (0%)	39	4 (10.3%)
Sweat	131	5 (3.8%)	55	0 (0%)	37	2 (5.3%)	39	3 (7.7%)
Neurologic symptoms	132	14 (10.6%)	55	0 (0%)	38	5 (13.2%)	39	9 (23.1%)
Coma	131	2 (1.5%)	55	0 (0%)	37	0 (0%)	39	2 (5.1%)
Agitation	131	10 (7.6%)	55	0 (0%)	37	2 (5.4%)	39	8 (20.5%)
Dysarthria	130	2 (1.5%)	54	0 (0%)	37	0 (0%)	39	2 (5.1%)
Muscular hypertonia	131	4 (3.1%)	55	0 (0%)	37	0 (0%)	39	4 (10.3%)
Mydriasis	131	1 (0.8%)	55	0 (0%)	37	0 (0%)	39	1 (2.6%)
Myosis	131	3 (2.3%)	55	0 (0%)	37	1 (2.7%)	39	2 (5.1%)
Paresthesia (general)	131	3 (2.3%)	55	0 (0%)	37	1 (2.7%)	39	2 (5.1%)
Lack of coordination	131	4 (3.1%)	55	0 (0%)	37	0 (0%)	39	4 (10.3%)
Visual disturbances	131	2 (1.5%)	55	0 (0%)	37	1 (2.7%)	39	1 (2.6%)
Dizziness	131	1 (0.8%)	55	0 (0%)	37	1 (2.7%)	39	0 (0%)
Headache	132	2 (1.5%)	55	0 (0%)	38	2 (5.4%)	39	0 (0%)
Anxiety	131	1 (0.8%)	55	0 (0%)	37	0 (0%)	39	1 (2.6%)
**ORL symptoms**	**131**	**9 (6.9%)**	**55**	**0 (0%)**	**38**	**1 (2.7%)**	**39**	**8 (20.5%)**
Hypersalivation	131	9 (6.9%)	55	0 (0%)	37	1 (2.7%)	39	8 (20.5%)
Tearing	130	0 (0%)	54	0 (0%)	37	0 (0%)	39	0 (0%)
Rhinorrhea	131	3 (2.3%)	55	0 (0%)	37	0 (0%)	39	3 (7.7%)
**Cardiovascular signs**	**132**	**63 (47.7%)**	**55**	**0 (0%)**	**38**	**31 (81.6%)**	**39**	**32 (82.1%)**
Hypertension	86	25 (29.1%)	27	0 (0%)	30	14 (46.7%)	29	11 (37.9%)
Hypotension	86	4 (4.7%)	27	0 (0%)	30	0 (0%)	29	4 (13.8%)
Tachycardia	125	28 (22.4%)	50	0 (0%)	37	22 (59.5%)	38	6 (15.8%)
Bradycardia	125	20 (16%)	50	0 (0%)	37	0 (0%)	38	20 (52.6%)
**Pulmonary signs**	**132**	**20 (15.2%)**	**55**	**0 (0%)**	**38**	**1 (2.6%)**	**39**	**19 (48.7%)**
Polypnea	33	15 (45.5%)	5	0 (0%)	8	0 (0%)	20	15 (75%)
Wrestling signs	127	3 (2.3%)	51	0 (0%)	38	0 (0%)	38	3 (7.9%)
Respiratory distress	127	3 (2.4%)	51	0 (0%)	37	0 (0%)	38	3 (7.9%)
Acute respiratory failure	126	6 (4.8%)	53	0 (0%)	37	0 (0%)	36	6 (16.7%)
Bronchospasm	131	1 (0.8%)	55	0 (0%)	37	0 (0%)	39	1 (2.6%)
Bronchial congestion	132	3 (2.3%)	55	0 (0%)	38	1 (2.6%)	39	2 (5.1%)
**Uro-digestive manifestations**	**131**	**20 (15.3%)**	**55**	**0 (0%)**	**38**	**6 (16.2%)**	**39**	**14 (35.9%)**
Nausea	130	14 (10.8%)	54	0 (0%)	37	3 (8.1%)	39	11 (28.2%)
Vomiting	130	12 (9.2%)	54	0 (0%)	37	3 (8.1%)	39	9 (23.1%)
Abdominal pain	130	10 (7.7%)	54	0 (0%)	37	3 (8.1%)	39	7 (17.9%)
Meteorism	130	4 (3.1%)	55	0 (0%)	37	1 (2.7%)	39	3 (7.9%)
Urinary retention	131	2 (1.5%)	55	0 (0%)	37	0 (0%)	39	2 (5.1%)
Priapism	131	0 (0%)	55	0 (0%)	37	0 (0%)	39	0 (0%)
**Cholinergic syndrome**	**132**	**75 (56.8%)**	**55**	**7 (12.7%)**	**38**	**36 (94.7%)**	**39**	**32 (82.1%)**
**Adrenergic syndrome**	**132**	**68 (51.5%)**	**55**	**3 (5.5%)**	**38**	**37 (97.4%)**	**39**	**28 (71.8%)**

Paraclinical investigations included three chest radiography, 71 electrocardiography and 62 blood sample tests (Table 4 shows the blood tests abnormalities recorded). Only one chest radiography showed alveolar syndrome. The analysis of the electrocardiogram record found sinus rhythm in all patients without sinus or ventricular electric dysfunction.

**Table 4.** Paraclinical investigations recorded in the general population and according to the grade of envenomation.

	Overall population		Grade 0-I		Grade II-III	
Parameter	Nb	Result	Nb	Result	Nb	Result
Biologic exams	132	62 (47%)	55	18 (32.7%)	77	44 (57.1%)
Biologic abnormalities	62	49 (79%)	18	12 (66.7%)	44	37 (84.1%)
Hematocrit (%)	58	38 (36-40)	17	39 (36.1-40)	41	38 (36-39)
Leukocytosis	58	7 (12.1%)	16	0 (0%)	42	7 (16.7%)
Elevated eosinophilia	56	19 (33.9%)	16	5 (31.3%)	40	14 (35%)
Thrombocytopenia	58	1 (1.7%)	16	0 (0%)	42	1 (2.4%)
Prothombin time < 60%	50	10 (20%)	14	1 (7.1%)	36	9 (25%)
Fibrinogen (g/L)	6	2.6 (2.4-2.8)	2	2.6 (2.6-2.7)	4	2.6 (2.3-2.7)
Protidemia (g/L)	56	73.9 (70-78)	16	74.3 (69-78.3)	40	73.5 (70.7-77.3)
Sodium (mmol/L)	59	139 (137-141)	16	138 (137-139)	43	139 (138-141)
Potassium (mmol/L)	57	3.8 (3.5-4.2)	14	3.9 (3.8-4.4)	43	3.8 (3.4-4.1)
Hypokalemia	57	12 (21.1%)	14	1 (7.1%)	43	11 (25.6%)
Metabolic acidosis	59	22 (37.3%)	16	2 (12.5%)	43	20 (46.5%)
Calcium (mmol/L)	57	2.45 (2.3-2.6)	16	2.39 (2.3-2.4)	41	2.5 (2.4-2.6)
Hypoglycemia	55	6 (10.9%)	15	1 (6.7%)	40	5 (12.5%)
Hyperglycemia	55	13 (23.6%)	15	1 (6.7%)	40	12 (30%)
Urea nitrogen (mmol/L)	57	4.1 (3.4-5)	16	3.7 (3.2-4.6)	41	4.1 (3.4-5)
Creatinine (µmol/L)	56	45.9 (37.2-57)	16	44.5 (37-55.5)	40	46 (37.3-57.3)
Bilirubin (mmol/L)	30	5 (2-6)	7	5 (4-5)	23	5 (3-6)
Elevated ASAT	39	1 (2.6%)	8	0 (0%)	31	1 (3.2%)
Elevated ALAT	38	2 (5.3%)	8	0 (0%)	30	2 (6.7%)
Elevated GGT	36	1 (2.8%)	8	1 (12.5%)	28	0 (0%)
Elevated AP	37	3 (8.1%)	8	0 (0%)	29	3 (10.3%)
Elevated lipase	3	1 (33.3%)	0	-	3	1 (33.3%)
Elevated troponin	18	8 (44.4%)	3	0 (0%)	15	8 (53.3%)
Elevated BNP	8	2 (25%)	1	0 (0%)	7	2 (28.6%)
Elevated myoglobin	5	2 (40%)	2	0 (0%)	3	2 (66.7%)
Elevated lactic acid	13	8 (61.5%)	0	-	13	8 (61.5%)

ALAT: alanine aminotransferases; ASAT: aspartate aminotransferases; GGT: gamma-glutamyltranspeptidase; AP: alkaline phosphatase; BNP: brain natriuretic peptide.

**Figure 5.**
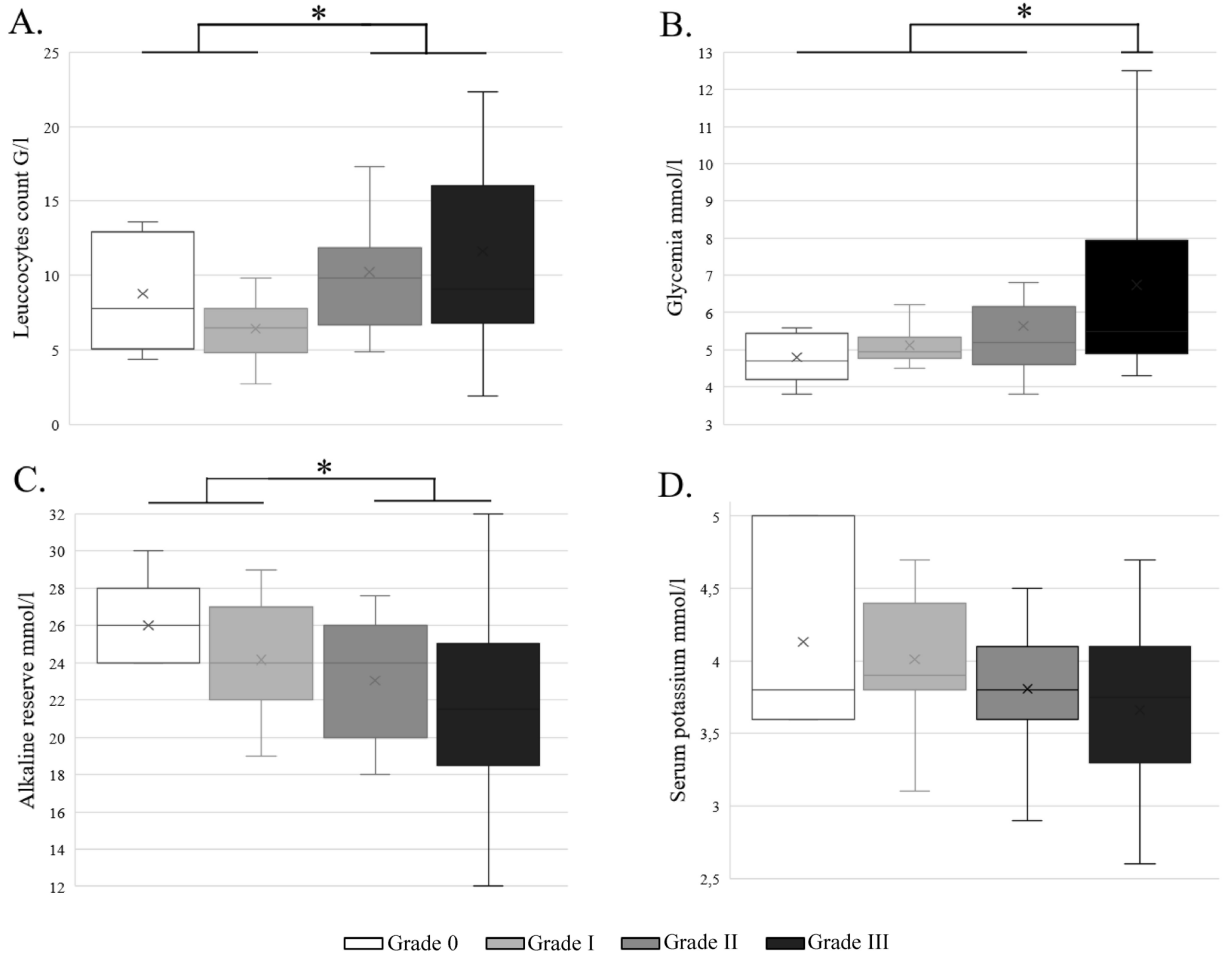
Blood sample results according to the grade of envenomation: **(A)** leukocyte count, **(B)** glycemia, **(C)** alkaline reserve and **(D)** serum potassium. *p < 0.05.

Leukocytosis was dosed in 58 cases (93%) (Figure 5A). The mean leukocytes count was 10 ± 6.3 G/L (extremes 1.9-42.2 G/L). Leukocytosis was significantly associated with general envenomation (p = 0.03). Glycemia was dosed in 55 cases (88%) (Figure 5B). The mean glycemia dosage was 6 ± 2.5 mmol/L (extremes 3.6-18.6 mmol/L). Hyperglycemia was significantly associated with Grade III envenomation (p = 0.02). Alkaline reserve was dosed in 59 cases (90%) (Figure 5C). The mean alkaline reserve dosage was 22.7 ± 4.1 mmol/L (extremes 12-32 mmol/L). A decrease in alkaline reserve was recorded in 22 patients (37%). It was significantly associated with Grade II and III envenomation (p = 0.02). Serum potassium was dosed in 57 cases (87%) (Figure 5D). It was 3.8 ± 0.6 mmol/L (extremes 1.9-5.0 mmol/L). Hypokalemia was found in seven patients (12%). The rate of hypokalemia increased with the grade of envenomation without statistical significance (p = 0.056). Lactic acid was dosed in 13 cases (20%). The mean lactic acid dosage was 3.3 ± 2.3 mmol/L (extremes 1.0-8.8 mmol/L). Hyperlactatemia was diagnosed in eight patients (61%). All of them were Grade II or III envenomation. 

### Therapeutic features and outcome

Ninety patients (68%) received a symptomatic treatment. Oxygen therapy has been administered to four patients (5%) and only one patient (0.8%) required mechanical ventilation. Analgesics were prescribed to 88 patients (67%). The prescribed analgesics were Class I, II, and III in 83 (66%), 11 (9%), and 13 patients (10%) respectively. There was no significant relationship between the class of analgesic used and the grade of envenomation. No use of prazosin or antivenom was recorded. 

Eighty-six patients (64%) received ambulatory treatment and 46 (36%) required a hospitalization. Outpatients were divided into 11 (12%) Grade 0, 32 (38%) Grade I, 24 (29%) Grade II, and 18 (21%) Grade III envenomation. The duration of medical management in the ED was 4.2 ± 3.3 hours, without statistical difference between grades of envenomation. Inpatients were divided into 4 (9%) Grade 0, 7 (15%) Grade I, 14 (30%) Grade II, and 21 (46%) Grade III envenomation. The hospital length of stay ranged between 1 and 14 days and was less than three days in 38 patients (82%). Hospitalization in intensive care unit was required for seven cases (six Grade III and one Grade II envenomation) and the hospitalization in a pediatric ward was required for nine patients (three Grade III, five Grade II, and one Grade 0 envenomation). No patient presented sequelae at the hospital discharge. No death was recorded during the study period.

**Figure 6.**
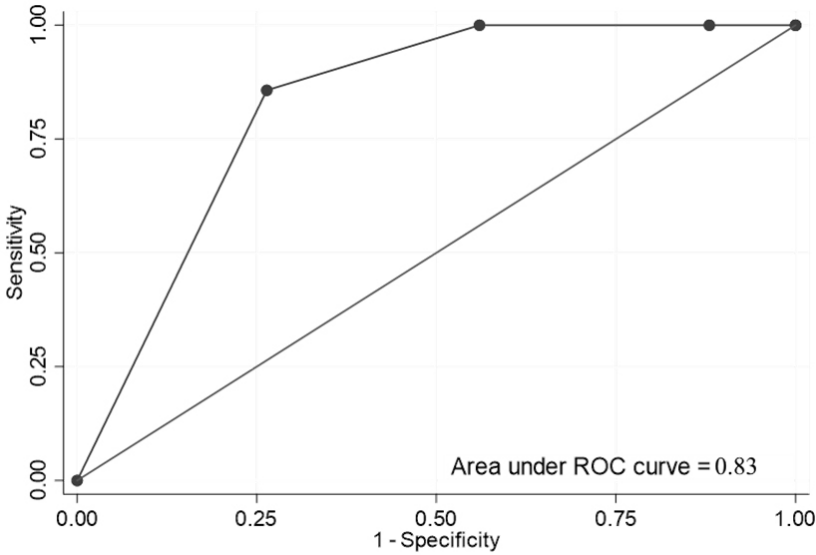
Receiver operating characteristic (ROC) curve showing the link between the grade of envenomation and a PRIMS II score ≥ 5 and (AUC: 0.83).

At the hospital discharge, the worst poisoning severity score (PSS) and the worst pediatric risk of mortality (PRISM) score were assessed. PSS score was divided into 21 (16%) PSS 0, 68 (52%) PSS 1, 36 (27%) PSS 2, and 7 (5%) PSS 3. All patients with PSS score of 3 were Class III envenomation. The mean PSS increased with the envenomation grade (p < 0.001). PRIMS was divided into 80 (61%) PRISM 0, 50 (38%) PRISM between 1 and 9, and 2 (1%) PRISM higher than 10. All patients with PRISM score strictly higher than 6 were Class III envenomed (Figure 6). The mortality rate predicted with PRISM score at 10 was 4.1%. 

The worst PRISM score recorded was 17. It was a Grade III envenomation, 11 years old boy with blood pressure at 202/135 mmHg (12 points), serum potassium at 3.5 mmol/L (1 point) and glycemia at 12.5 mmol/L (4 points). Predicted mortality was 22% and 12.8% when the score is adapted for age. He was hospitalized in intensive care unit for three days. The second worst PRISM score was at 16 points. It was a Grade III envenomation, 8 years old girl with heart rate at 60 beat per minute (4 points), serum potassium at 2.6 mmol/L (5 points), glycemia at 18.6 mmol/L (4 points) and alkaline reserve at 12 mmol/L (3 points). She was hospitalized in the pediatric unit for onbe day. Predicted mortality was 18.7% and 8.4% when the score is adapted for age. The longest intensive care unit hospitalization was for a 7 years old boy with severe acute pancreatitis. This case was previously reported by Kallel et al. [30]. He had a PRISM score at 8.

## Discussion

This study shows that the rate of scorpion stings in pediatric patients is closely correlated to some climatic conditions. It can be responsible for several clinical pictures ranging from local pain to severe organ dysfunction. Despite the severity of this pathology and the need for intensive care in some cases, all patients recovered well and, no death was recorded.

In the present study, we recorded eight cases per year in the average. As the study is monocentric, we are convinced that the incidence rate of pediatric scorpionism is underestimated in French Guiana. Indeed, in many cases, patients do not consult their physicians after the sting [9]. It is noteworthy that in French Guiana, the traditional pharmacopoeia is widely used. It is composed of vegetal, animal and mineral substances, known and relayed by experience. In either Amerindian or Bushinengues communities, traditional medicine is practiced, on a daily basis, by shamans or traditional doctors in whom native people have entire trust [31,32].

Around the world, deaths secondary to scorpion envenomation occurred mostly in patients under 15 years old [9]. Furthermore, preschool children present more complications than older ones, and represent the most vulnerable population [9]. The two cases of death previously reported in French Guiana were in this age range [16,17]. Thus, a better understanding of this pathology and an adapted medical care are mandatory to avoid new fatal cases.

Unless the scorpion is clearly seen when stinging, its description is often very subjective. Scorpion color and pincer size can be difficult to describe for untrained persons. Thus, no scorpion identification based on a visual description is relevant [33]. The French Guiana killer, *Tityus obscurus*, is brown when it is young and may be confused with many other scorpions. Once it reaches adulthood, it turns into black and visually similar to *Jaguajir pintoi kourouensis* [11]. Therefore, statistics and evaluation based on scorpion visual description should be performed with caution. In the present study, the scorpion was described as “big” (48%), “black” (60%), and “small pincer” (58%). In many cases, the scorpion was not captured and the identification was based only on the description given by the patient or by witnesses, so that, any conclusion about the responsible scorpion species would be interpretative.

The time of the sting is a key element in scorpion envenomation. This study suggests three specific periods when most of the stings occur. The first period is in the morning where children are stung when dressing up. The second period is around lunch time, when school children go home for lunch and take off their shoes. The third period is at sleeping time when children wear nightclothes. A fourth less significant time period is identified by the end of school day, with a similar reason to those for the midday. In these periods, children are stung because they are not aware or do not pay attention to the presence of the scorpion. In the other periods, stings occurred only during children’s activities and rarely during the sleeping time. It is noteworthy that scorpions can adapt to their environment and can live in human habitations [2,13]. We also noted that most of the stings occurred in coastal cities and at home. A more prudent behavior at home, during at-risk periods could decrease efficiently the number of stings. It is interesting to emphasize that periods of activity of children and those of scorpion are different. Scorpions are essentially active during the first five hours of the night [34]. This suggests that stings are due to a defensive behavior when scorpions are disturbed. 

The correlation between the stings rate and the climatic conditions is obvious. The rainy weather was correlated with scorpion stings in other Amazonian studies [3,4]. However, this parameter does not explain why scorpions moved to urbanized areas when it is raining. Further studies identified fluvial water level as the most significant variable [35]. We argue that the fluvial water level leads the scorpion to move to dryer places. Consequently, urban areas represent a safer environment, as they are non-floodable. At this time, scorpion can cross human’s path and sting them [36]. This can partially explain why stings occurrence is higher at home than in forest. In the present work, we found similar results with a significant correlation between the rate of stings and the river level variation. Thus, urban area represents safe places for scorpions when the river level rises. It is noteworthy that the other climatic conditions did not show significant impact on the rate of scorpion stings. The impact of monthly river level variation on the epidemiology of scorpion sting was not described yet. We think that scorpions do not have to flee to seek a new refuge when rivers are stagnant. By the way, scorpion sting can occur also during the dry season, when children wear less clothes and open shoes. 

Clinical symptoms range from local pain to cardiovascular, respiratory, neurologic, or digestive symptoms [6]. In the present study, local symptoms were present in 47% of patients. However, the most common symptom of envenomation was abnormal heart rate (61% of cases of Grade II or III envenomation). This can be explained by the scorpion venom action on the adrenergic and cholinergic pathways [19]. The venom spreads in the organism in less than 1 hour [37] and most of patients come to hospital between the second and third hour after being injured. This is why researching abnormal heart beat seems the most relevant sign to determine if there is a severe envenomation. The most serious envenomations are also associated to cholinergic symptoms such as hypersecretion, lacrimation, rhinorrhea, and abdominal pain related to an over-secretion of digestive enzymes. Digestive or neurologic symptoms are also linked to major envenomation [38-41]. In this study, these symptoms were linked to Grade III envenomation too. Numerous abnormalities are highlighted on blood samples analysis. Some of them are related to the activation of the adrenergic pathway. This is the case of hyperglycemia, hypokalemia and metabolic acidosis. Hyperglycemia was found to be linked to unfavorable outcome and cardiac dysfunction in patients with scorpion sting [42,43]. So, the capillary glycemia should be checked as soon as possible at the emergency department. This parameter can provide a quick orientation on the severity of the envenomation. In our hospital, capillary glycemia at admission is part of the standard care in the emergency department. The presence of hyperglycemia and hypokalemia may suggest a dysregulation of insulin or sodium-glucose cotransporters, and Na/K exchange pumps, as described with other scorpions [44]. Other blood analysis abnormalities might be related to cholinergic pathway activation with hypersecretory action including pancreatic enzymes. This condition was observed in one patients who developed acute necrotic pancreatitis [30]. Indeed, pancreatitis is a rare but serious complication that can lead to death [45]. Lactic acidosis is observed in many patients with general symptoms of envenomation. This biological marker reflects cellular anoxia. It is generally associated to an increase in cardiac markers with a moderate elevation of troponin and a minimal elevation of BNP. This elevation can be associated to left ventricular dysfunction [46] and echocardiography is the standard investigation to search for this classical complication in scorpion envenomed patients [47]. In our study, no patient had signs of cardiac dysfunction and no echocardiography was performed.

The management of those patients is principally based on pain killers use as the patient’s suffering is at the forefront of the envenomation. It presents as an electrical discharge going up along the stung limb. Its intensity is subject to inter-individual variability making difficult to link the level of pain to the grade of envenomation. As a result, it is impossible to propose a systematic analgesic schema. A recent study showed an equal effect between cold pack and paracetamol for the management of local pain, but a better analgesia with local lidocaine [48]. In the present study, class I, II, and III analgesics were used in 68% of cases. 

Neither antivenom nor prazosin is currently available in French Guiana. Indeed, scorpion antivenom is difficult to synthesize due to the large number of scorpions in FG. Furthermore, its price and shelf life make it an expensive product [49]. Thus, it seems unnecessary to acquire it for such relatively safe scorpion envenomation. Concerning the prazosin, its benefit is debated [48]. Indeed, even though our patients mainly presented cardio-circulatory disorders, without neurological or pulmonary damages, the cost efficiency of prazosin can be valuable. Nevertheless, this treatment has no authorization for use in case of scorpion envenomation in our country. This limits our possibility of management in envenomed children without going through a temporary administrative authorization.

The PRISM score seems relevant to discriminate the different Grades in case of scorpion envenomation. However, it is not relevant to predict mortality in scorpion envenomed children in French Guiana. In the other hand, the PSS did not seem relevant in case of scorpion envenomation. The PSS over estimates the severity of the envenomation because affected patients are often very painful and major pain alone can class patient with PSS at 3. However, if the patient's pain is not considered, the PSS tends to underestimate the severity of the envenomation.

The reversibility of the symptoms is common after scorpion sting. In our patients, all the effects of the sting had recovered and there was no complication after hospital discharge. In all cases, patients returned home safely after few hours of monitoring or after hospitalization.

## Conclusion

Scorpion envenomation in French Guiana is related to the activity of children. A simple preventive behavior such as checking shoes and clothes before wearing could reduce significantly the number of stings. This study highlights a climatic impact on scorpion activities and stings in the Amazon region where the rate and the severity of envenomation is strongly related to the rivers water level and to the absolute rivers level variation. After scorpion sting, the rate of general symptoms decreases with age. The peak of risk involves children under 3 years old. The clinical and biological presentations are rarely serious. They include local pain associated to adrenergic and/or cholinergic symptoms. In view of the potential risks and frequency of Grade II and III envenomation, children under 6 years old should be assessed by a pediatrician or in an emergency department. The PRISM score seem relevant to grade envenomation. However, more specific triage and assessment is needed to improve hospital and pre-hospital triage, reduce the number of helicopter transports and thus the number of hospitalizations and ultimately the overall cost of managing scorpion envenomation.
